# Supplementary material for: Fire and grazing determined grasslands of central Madagascar represent ancient assemblages
Source: Proc Biol Sci. 2020 May 13;287(1927):20200598. doi: 10.1098/rspb.2020.0598 (PMC7287345; doi:10.1098/rspb.2020.0598)
Supplement: Supplementary Material - Fire and grazing determined grasslands of central Madagascar represent ancient assemblages [file rspb20200598supp1.pdf]

Supplementary Materials

Title: Fire and grazing determined grasslands of central Madagascar represent ancient assemblages.

Authors: Cédrique L. Solofondranohatra[1,2], Maria S. Vorontsova[3], Gareth P. Hempson[4], Jan Hackel[3], Stuart Cable[2,5], Jeannoda Vololoniaina[1] and Caroline E. R. Lehmann[4,6,7]

[1]Laboratoire de Botanique, Département de Biologie et Ecologie Végétales, Faculté des Sciences, Université d'Antananarivo, Antananarivo, Madagascar

[2]Kew Madagascar Conservation Centre, Antananarivo, Madagascar

[3]Comparative Plant and Fungal Biology, Royal Botanic Gardens, London, United Kingdom

[4]Centre for African Ecology, School of Animal and Plant Sciences, University of the Witwatersrand, Johannesburg, South Africa

[5]Conservation Science, Royal Botanic Gardens, London, United Kingdom

[6]School of GeoSciences, The University of Edinburgh, Edinburgh, United Kingdom

[7]Tropical Diversity, Royal Botanic Garden Edinburgh, Edinburgh, United Kingdom

Journal: Proceedings of the Royal Society B.

Doi: 10.1098/rspb. 2020.0598

Supplementary Figures 1 – 4

Supplementary Tables 1 – 3

References

Supplementary Figure 1: *Map of Madagascar depicting limits of grasslands*. The central ecoregion as per Humbert (1955) is shaded grey. Plateau grassland- wooded grassland mosaic distribution as per Moat and Smith (2007) is shaded in green. Locations of study sites are shown as are names of regions where sampling was undertaken.

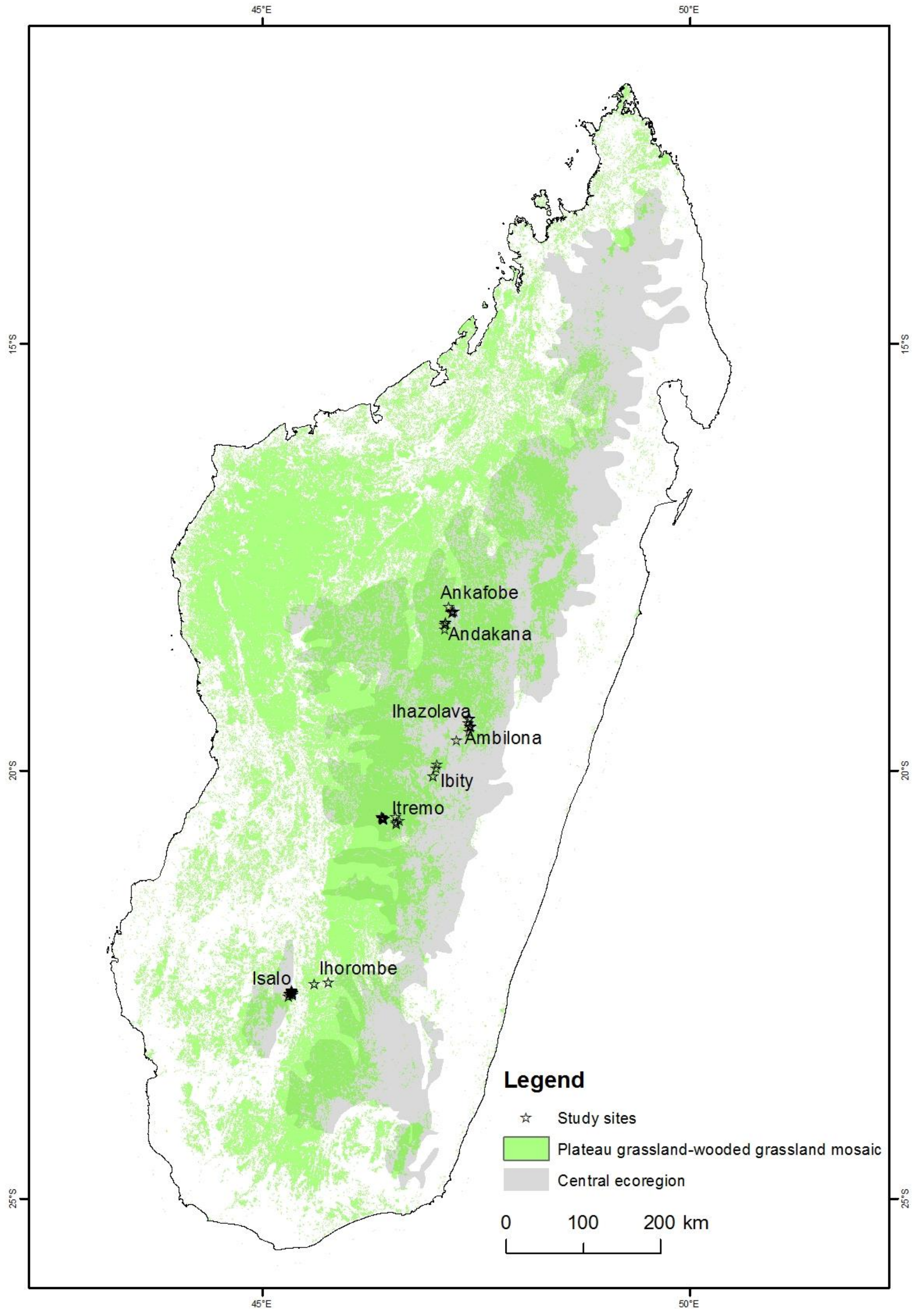

Supplementary Figure 2: *Environmental variables distribution* (A) Histograms of mean annual precipitation (Bio_12, Worldclim Global Climate Data version) and percent sand in the top 10 cm soil (Harmonised World Soils Database) across the central ecoregion as mapped by Humbert (1955). (B) Across the 71 study sites, histograms of mean annual precipitation, percent sand in the top 10 cm soil, and distance to road.

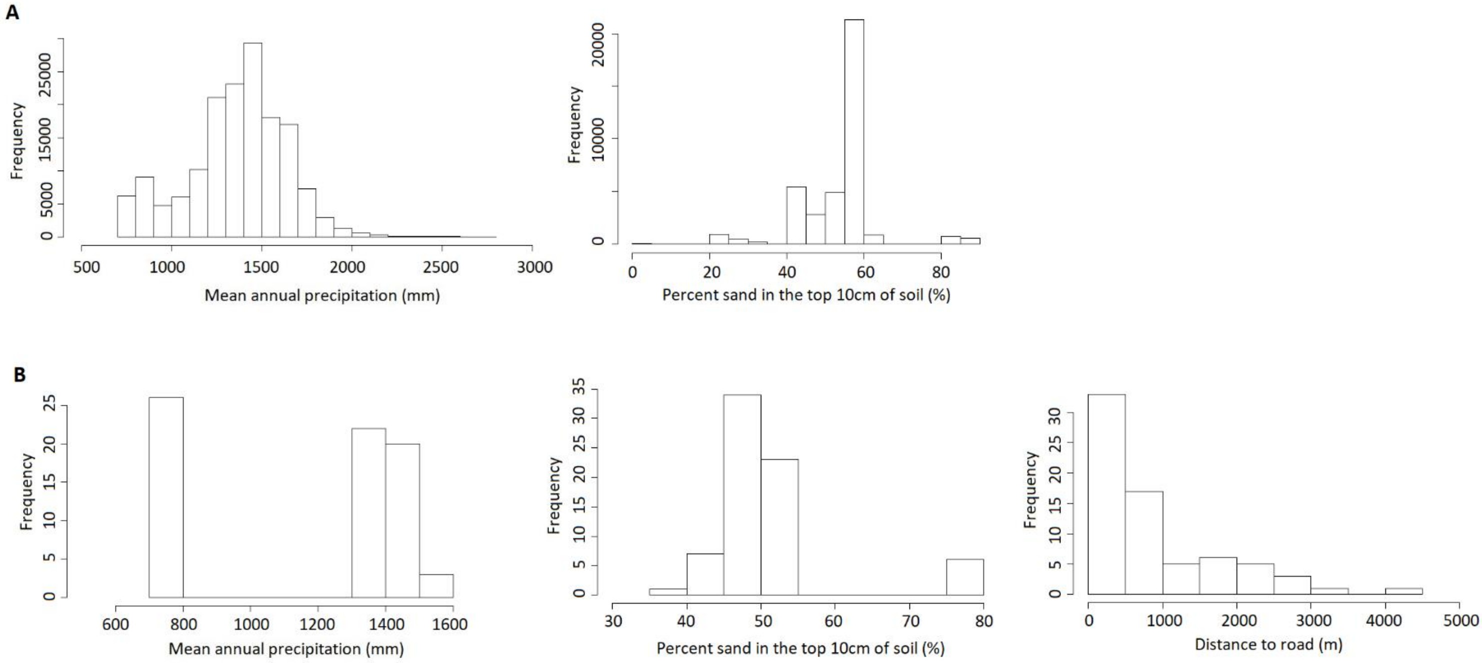

Supplementary Table 1: *Table of all grass species encountered.* Table describes: 1) endemicity; 2) number of sites where species were found; 3) maximum number of occurrences per site (out of a maximum of 21); 4) rarity as defined and described in the methods of the main text; and, 5) assemblage group (1 or 2). Assemblage groups are based on residual correlations values between pairs of species as a product of the generalized linear latent variable model described in the main methods. The analysis used only 41 common species and post-hoc assemblage group were assigned to the rare species.

**Genera**	**Species**	**Endemic**	**Number of sites of occurrence (out of 71)**	**Maximum number of occurrence per site (out of 21)**	**Rare**	**Assemblage group**
Agrostis	*elliotii*	yes	1	1	yes	2
Alloteropsis	*semialata*	no	4	5	yes	2
Andropogon	*itremoensis*	yes	1	4	yes	2
Andropogon	*trichozygus*	yes	1	23	no	NA
Aristida	*rufescens*	no	12	20	no	1
Aristida	*similis*	yes	2	5	yes	2
Aristida	*tenuissima*	yes	21	24	no	2
Axonopus	*compressus*	no	4	7	no	1
Brachiaria	*arrecta*	no	1	17	no	NA
Brachiaria	*subrostrata*	yes	3	14	no	1
Brachypodium	*madagascariense*	yes	1	1	yes	2
Chrysopogon	*serrulatus*	no	11	20	no	2

Craspedorhachis	*africana*	no	18	15	no	NA
Ctenium	*concinnum*	no	5	16	no	2
Cymbopogon	*caesius*	no	4	5	yes	2
Cynodon	*dactylon*	no	12	20	no	1
Cyrtococcum	*deltoideum*	yes	1	1	yes	1
Digitaria	*ciliaris*	no	4	7	no	1
Digitaria	*debilis*	no	5	5	yes	NA
Digitaria	*longiflora*	no	25	21	no	1
Digitaria	*pseudodiaginalis*	no	6	6	no	2
Digitaria	*thouaresiana*	no	1	2	yes	NA
Eleusine	*indica*	no	9	18	no	1
Eragrostis	*atrovirens*	no	6	19	no	1
Eragrostis	*chapelieri*	no	1	6	no	NA
Eragrostis	*lateritica*	yes	17	15	no	1
Eragrostis	*racemosa*	no	6	13	no	1
Eragrostis	*tenella*	no	2	5	yes	1
Eragrostis	*tenuifolia*	no	4	6	no	NA
Eulalia	*villosa*	no	1	4	yes	NA
Festuca	*camusiana*	yes	1	1	yes	2
Heteropogon	*contortus*	no	9	21	no	1
Hyparrhenia	*newtonii*	no	19	15	no	2

Hyparrhenia	*rufa*	no	18	21	no	1
Imperata	*cylindrica*	no	5	5	yes	NA
Loudetia	*filifolia*	no	11	21	no	2
Loudetia	*simplex*	no	58	25	no	2
Melinis	*minutiflora*	no	5	3	yes	2
Melinis	*repens*	no	4	5	yes	2
Microchloa	*kunthii*	no	7	8	no	1
Oplismenus	*burmanii*	no	2	1	yes	2
Panicum	*cinctum*	yes	12	13	no	2
Panicum	*ibitense*	yes	4	5	yes	2
Panicum	*perrieri*	yes	3	2	yes	2
Panicum	*subhystrix*	yes	5	5	yes	2
Panicum	*umbellatum*	yes	23	21	no	1
Paspalum	*scrobiculatum*	no	16	18	no	1
Pennisetum	*pseudotriticoides*	yes	5	13	no	2
Pogonarthria	*squarosa*	no	2	2	yes	2
Schizachyrium	*brevifolium*	no	3	16	no	NA
Schizachyrium	*exile*	no	7	12	no	1
Schizachyrium	*sanguineum*	no	49	22	no	2
Setaria	*pumila*	no	14	20	no	1
Setaria	*sphacelata*	no	2	2	yes	1

Sporobolus	*centrifugus*	no	21	18	no	1
Sporobolus	*paniculatus*	no	3	7	no	1
Sporobolus	*piliferus*	no	1	1	yes	2
Sporobolus	*pyramidalis*	no	5	20	no	1
Stenotaphrum	*oostachyum*	yes	6	15	no	1
Stenotaphrum	*unilaterale*	yes	1	4	yes	1
Styppeiochloa	*hitchcockii*	yes	1	1	yes	2
Trachypogon	*spicatus*	no	45	25	no	2
Tricanthecium	*brazzavillense*	no	2	5	yes	2
Tricholaena	*monache*	no	3	5	yes	2
Tristachya	*humbertii*	yes	4	17	no	1
Tristachya	*isalensis*	yes	5	9	no	2
Urelytrum	*agropyroides*	no	6	19	no	2

Supplementary Table 2: *Description of five measured traits alongside collection method, related function and literature references.* The five traits are: 1) leaf table height (H_{LT}, cm); 2) leaf thickness (LT, cm); 3) leaf size: leaf width to leaf length ratio (LW/LL); 4) growth form (mat forming, rambling, caespitose); and, 5) bulk density (BD, g/cm^3)]).

Traits	Collection method	Related function	References
Leaf table height (H_{LT}, cm)	The height visually estimated to correspond to the c. 80th quantile of leaf biomass was measured on three individuals per species.	Plant height is a key functional trait with consequences for light competition in frequently burnt environment. Tall grasses are effective competitors for light, often associated with high total biomass and are more flammable which reinforce a fire feedback to increase flammability. Tall grasses are "fire resistors and grazer avoiders". Short grasses have low proportion of stem material and are relatively higher-quality forage.	Westoby, 1998 ; Dìaz et al., 2016; D'Antonio & Vitousek, 1992; Rossiter et al., 2003; Archibald et al., 2019 ; Hempson et al., 2015
Leaf thickness (LT, cm)	Leaf thickness was measured on three fully expanded leaves on each of three individuals per species.	Leaf thickness is related to its toughness and digestibility. Toughness is among the most important mechanical attributes	Theron and Booysen, 1966;

		influencing grazing. Thick, tough leaves are less digestible to herbivores. They are hypothesized to have high carbon content to make grasses more flammable. Thinner soft leaves are more palatable and attract grazers.	Coley, 1983; Wilson et al., 1983;
Leaf size: leaf width to leaf length ratio (LW/LL)	Leaf width and length were measured on the same three leaves per individual per species for leaf thickness measurement.	Large versus small leaves are grazing and fire attraction traits respectively. Large leaves are more palatable and preferred by grazers by reducing foraging time. Small leaves arranged in an aerated canopy ignite easily and burn intensely, i.e. more flammable.	Stobbs, 1973; Archibald et al., 2019; Schwilk, 2015
Growth form (mat forming, rambling, caespitose)	Growth form were recorded for each species.	Mat-forming habit with culms growing laterally is a grazing adaptation trait. With this growth form, most of the meristematic tissues are kept below grazing depth, allowing grasses to resist intense grazing. In contrast, caespitose grasses with erect culms can protect their	Hempson et al., 2015 ; Linder et al., 2018 ; Dìaz et al., 2007. Hempson et al., 2019 ; Archibald et al., 2019

		meristematic tissue from fire damage with intravaginal buds protected within basal leaf sheaths or underground, and tillers tightly clustered. Caespitose growth form can be associated with “generalist tolerators” and “avoiders” life histories as well. Rambling species are characterized by culms with an architecture in between prostrate and upright, which are better light competitor than mat-forming species but less than caespitose species.	
Bulk density (BD, g/cm^3)	Bulk density is the ratio between plant biomass and volume. It is calculated by dividing the total aboveground biomass by an estimate of the grass canopy volume. Volume was calculated using measures of the tuft basal diameter (D_B), leaf table height (H_{LT}) and leaf table diameter (D_{LT},	Species with high bulk density attract grazers with a high density of palatable leaves clustered in the canopy which promote grazing. Intermediate bulk density promotes fire spread with enough fuel to burn and sufficient air flow for combustion.	Hempson et al., 2019; Coughenour, 1985.

	diameter at H_{LT}). For caespitose grasses, volume (V) was calculated using the formula for a truncated cone: $V = \pi / 3 * H_{LT} * ((D_B / 2)^2 + (D_{LT} / 2)^2 + D_B * D_{LT})$. For mat-forming grasses, a square of the individual(s) was marked out using a spade, and the volume was calculated as a cube: $V = D_B * D_{LT} * H_{LT}$. Aboveground biomass was determined on three individuals per species by clipping, drying (at 60°C for 72 h) and weighing (using a scale with two decimal place scale) the parts of the individual for which the volume estimate was made.		

Supplementary Figure 3: *Histograms of residual correlations values, estimated from a generalized latent variable model for each species*. Model incorporates mean annual precipitation, presence/absence of fire, distance to road and a single latent variable. Values range from -1 to +1 and species with residual correlations ranging from -0.1 to +0.1 represent a lack of any association and were not classified into assemblages.

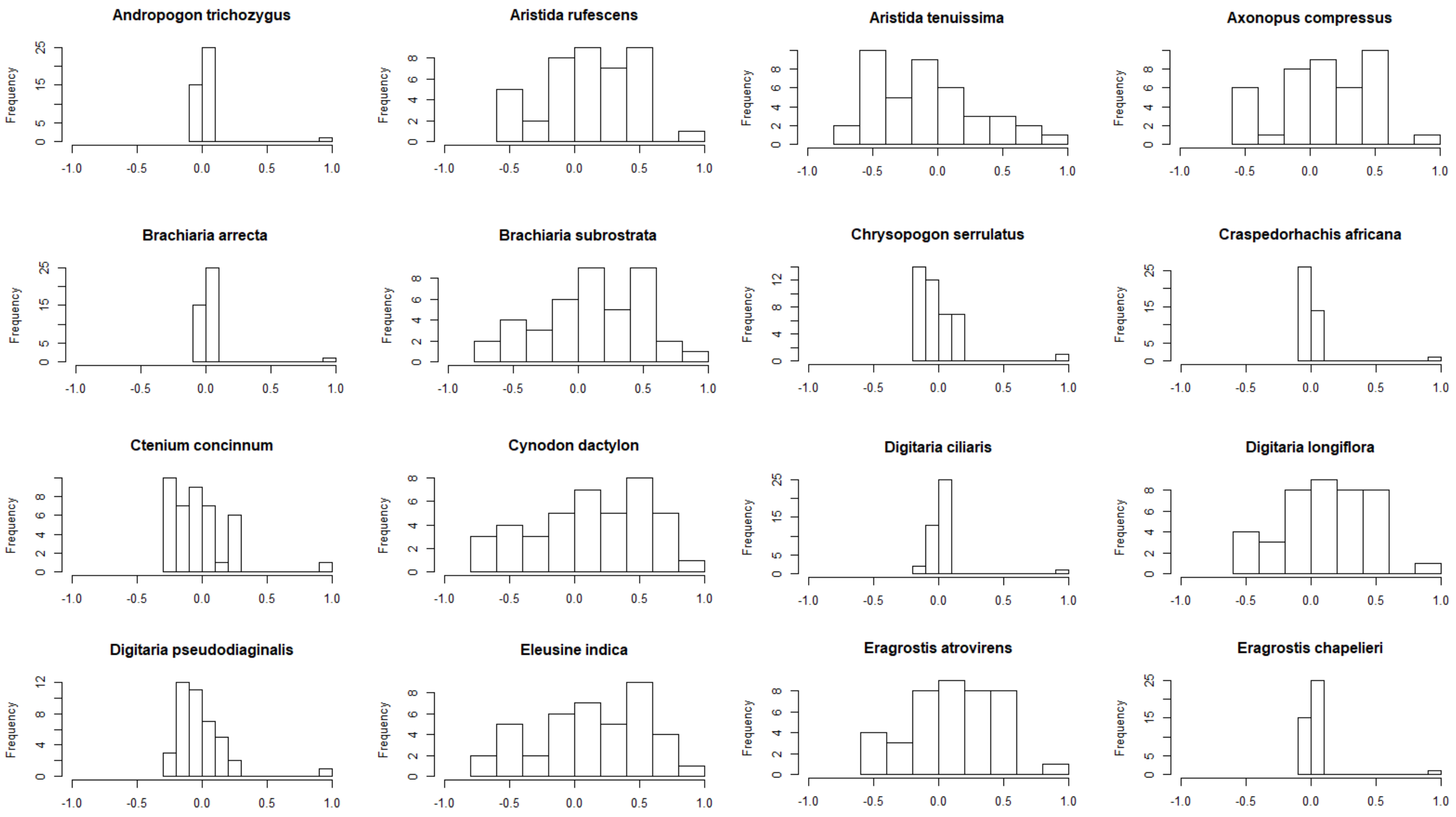

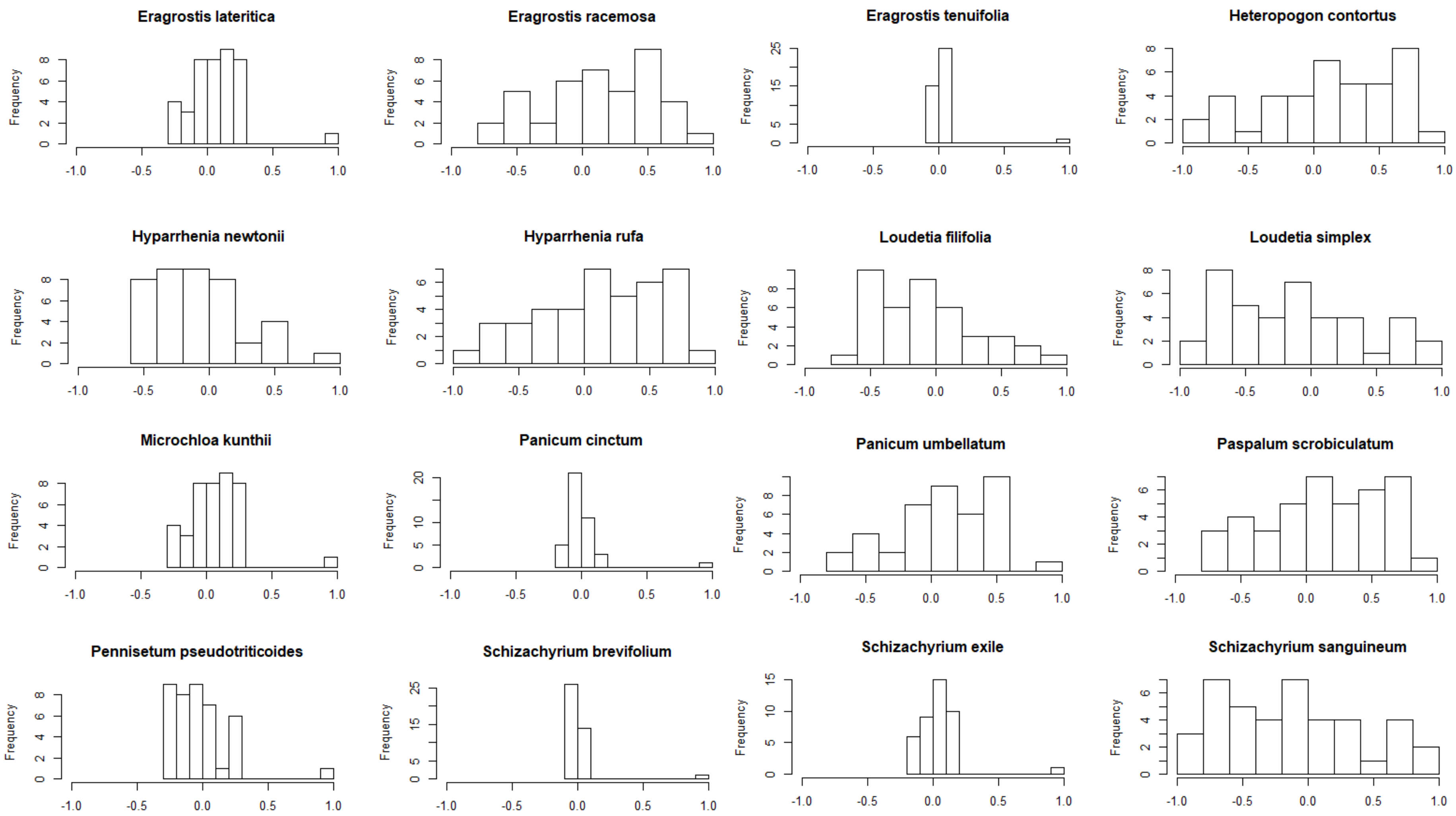
Eragrostis lateritica
Eragrostis racemosa
Eragrostis tenuifolia
Heteropogon contortus
Hyparrhenia newtonii
Hyparrhenia rufa
Loudetia filifolia
Loudetia simplex
Microchloa kunthii
Panicum cinctum
Panicum umbellatum
Paspalum scrobiculatum
Pennisetum pseudotriticoides
Schizachyrium brevifolium
Schizachyrium exile
Schizachyrium sanguineum
Frequency
-1.0 -0.5 0.0 0.5 1.0

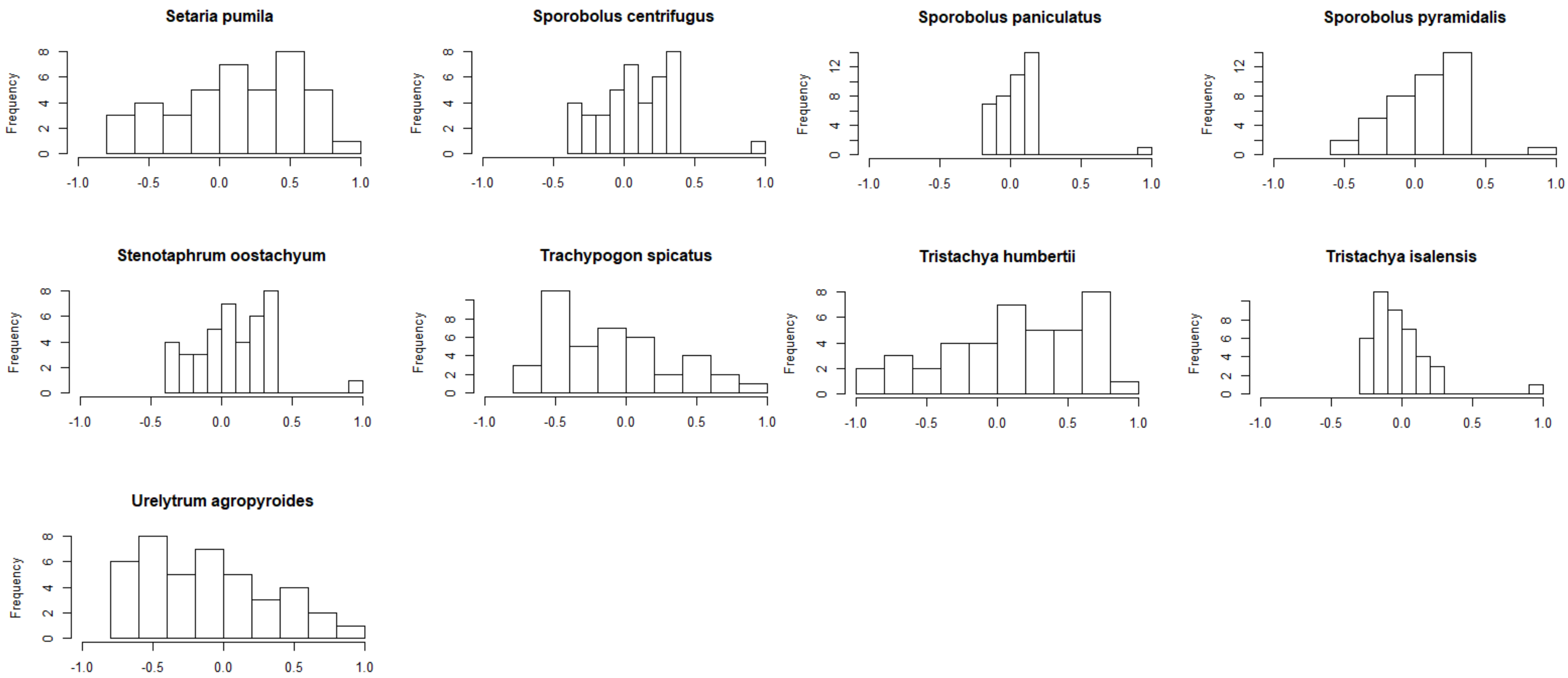
Setaria pumila
Sporobolus centrifugus
Sporobolus paniculatus
Sporobolus pyramidalis
Stenotaphrum oostachyum
Trachypogon spicatus
Tristachya humbertii
Tristachya isalensis
Urelytrum agropyroides
Frequency
-1.0
-0.5
0.0
0.5
1.0

Supplementary Table 3: *Table of Akaike Information Criterion (AIC) values derived from generalized latent variable models.* Values correspond to the different environmental covariates' association used in the models of grass species frequency data in addition to a single unobserved predictor (latent variable). AIC values were sorted from the lowest to the highest and the model with mean annual precipitation (MAP), distance to road, presence/ absence of fire was kept for interpretation.

**Environmental covariates used for the model**	**AIC values**
MAP + distance to road + presence/ absence fire	4904.07
MAP + distance to road + presence/ absence fire + percent sand	4906.25
MAP + presence/ absence fire	4923.8
MAP + distance to road	5011.67
MAP + distance to road + percent sand	5016.9
MAP	5040.02
MAP + percent sand	5043.26
distance to road + presence/ absence fire + percent sand	5168.96
presence/ absence fire + percent sand	5179.44
distance to road + presence/ absence fire	5193.85
presence/ absence fire	5199.39
distance to road	5348.65
distance to road + percent sand	5356.67
percent sand	5363.98
null model	5393.02

Supplementary Figure 4. Grass *species richness and phylogenetic diversity across assemblage group.* Assemblage groups (1 and 2) are based on residual correlations values between pairs of species as a product of the generalized linear latent variable model described in the main methods. No significant differences were found between species richness but phylogenetic diversity differed significantly between the two groups (GLM, $P < 0.001$).

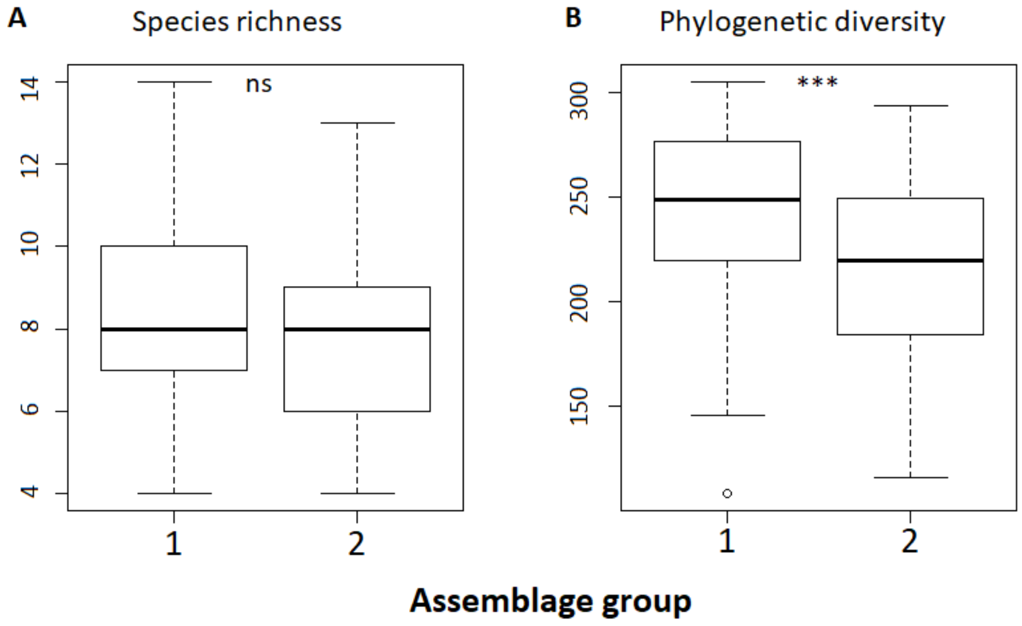

References

Archibald, S., Hempson, G.P., and Lehmann, C.E.R. (2019). A unified framework for plant life history strategies shaped by fire and herbivory. *New Phytol.* doi:10.1111/nph.15986

Coley, P.O. (1983) Herbivory and defensive characteristics of tree species in a lowland tropical forest. *Ecological Monographs* 53, 209–233.

Coughenour, M. B. (1985). Graminoid responses to grazing by large herbivores: adaptations, exaptations, and interacting processes. *Annals of the Missouri Botanical Garden*, 72, 852–863

D'Antonio, C. M. and Vitousek, P. M. (1992). Biological invasions by exotic grasses, the grass/fire cycle, and global change. *Annu. Rev. Ecol. Syst*. 23, 63–87

Dìaz, S., Kattge, J., Cornelissen, J. H. C., Wright, I. J., Lavorel, S., Dray, S. et al., (2016). The global spectrum of plant form and function. *Nature* 529: 167–171.

Dìaz, S., Lavorel, S., McIntyre, S. U. E., Falczuk, V., Casanoves, F., Milchunas, D. G. et al. (2007). Plant trait responses to grazing–a global synthesis. *Global Change Biology*, 13, 313–341.

FAO/IIASA/ISRIC/ISSCAS/JRC, 2009. Harmonized World Soil Database (version 1.2). FAO, Rome, Italy and IIASA, Laxenburg, Austria.

Fick, S.E. and Hijmans, R.J. (2017). Worldclim 2: New 1-km spatial resolution climate surfaces for global land areas. *Int. J. Climatol.* 37, 4302-4315.

Hempson, G. P., Archibald, S., Donaldson, J. E., and Lehmann, C. E. (2019). Alternate Grassy Ecosystem States Are Determined by Palatability–Flammability Trade-Offs. *Trends in ecology & evolution*, 34, 286–290.

Hempson, G.P., Archibald, S., Bond, W.J., Ellis, R.P., Grant, C.C., Kruger, F.J., Moxley, C., Owen-Smith, N., Peel, M.J.S., Smit, I.P.J. et al. (2015). Ecology of grazing lawns in Africa. *Biol. Rev.* 90, 979–994. doi:10.1111/brv.12145

Humbert, H. (1955). Les territoires phytogéographiques de Madagascar. *Ann. Biol.* 31, 439–448.

Linder, H. P., Lehmann, C. E. R., Archibald, S. A., Osborne, C. P., and Richardson, D. M. (2018). Global grass (Poaceae) success underpinned by traits facilitating colonization, persistence and habitat transformation. *Biol. Rev.* 93, 1125–1144. doi:10.1111/brv.12388

Rossiter, N. A., Setterfield, S. A., Douglas, M. M., Hutley, L. B. (2003). Testing the grass-fire cycle: alien grass invasion in the tropical savannas of northern Australia. *Diversity and Distributions* 9: 169–176.

Schwilk ,D.W. (2015) Dimensions of plant flammability. *New Phytol*. 206, 486–488. (doi:10. 1111/nph.13372)

Stobbs, T. H. (1973). The effect of plant structure on the intake of tropical pastures. I. Variation in the bite size of grazing cattle. *Crop and Pasture Science,* 24: 809–819.

Theron, E. P. and Booysen, P. de V. (1966). Palatability in grasses. *Proceedings of the Grassland Society of South Africa* 1, 111–120.

Westoby, M. (1998). A leaf-height-seed (LHS) plant ecology strategy scheme. *Plant and Soil* 199: 213–227.

Wilson, J. R., Brown, R. H. and Windham, W.R. (1983). Influence of leaf anatomy on the dry matter digestibility of C3, C4 and CJ/C4 intermediate types of Panicum species. *Crop Science*. 23, 141–146.
